# Advanced Materials & Methods for Heritage & Archaeology (Second Edition)

**DOI:** 10.3390/ma19153247

**Published:** 2026-07-31

**Authors:** Angela Calia, Mariateresa Lettieri

**Affiliations:** 1 CNR-ISPC (Italian National Research Council—Institute of Heritage Sciences), 73100 Lecce, Italy; 2 CNR-SPIN (National Research Council—Institute for SuPerconductors, INnovative Materials, and Devices), 84084 Fisciano, Italy

## 1. Introduction and Scope

Heritage Science has emerged as a vibrant, rapidly expanding domain where the arts, humanities, and hard sciences converge [1]. Cultural heritage assets are tangible anchors of human history. However, they are continuously subjected to the relentless forces of environmental degradation, climate change, and anthropogenic pollution [2]. To understand, preserve, and sustainably manage these invaluable assets, traditional methods, such as those reliant on invasive sampling and highly localized investigations are no longer sufficient. Modern archaeometry and diagnostics demand a deep physical, chemical, and structural understanding of the artifacts for a comprehensive historic-artistic knowledge [3,4], as well as to support modern preservation, which in turn widely intersects materials science [5]. Investigations also demand analytical tools and protocols that can safely highlight a material’s features and structural aspects of heritage assets with minimal or zero damage to their integrity. A profound paradigm shift has taken place in Heritage Science, evolving towards an advanced interdisciplinary and non-invasive or minimally invasive approach, also in the quantitative analytical domain [6,7].

Currently, the state of the art in Heritage Science revolves around three core objectives:

1. Minimizing diagnostic footprint by transitioning from destructive materials testing to non-destructive (NDT) or micro-destructive (MDT) testing protocols.

2. Optimizing analytical protocols by using high resolution, micro-destructive analytical technics and by replacing single-technique methods with integrated, multi-analytical pathways.

3. Broadening access to preservation tools by moving away from cost-prohibitive equipment toward affordable, field-deployable, in situ technologies for continuous monitoring.

This Special Issue, “Advanced Materials & Methods for Heritage & Archaeology (Second Edition),” compiles original research contributions deeply aligned with these objectives.

The published works explore the mechanical properties, composition, degradation pathways, and provenance of cultural assets, providing invaluable tools for their knowledge and for well-timed preventive conservation. By adapting advanced materials-testing protocols, non-destructive diagnostic systems, and innovative micro-analytical workflows to the delicate nature of historical artifacts, these studies attempt to close critical methodological gaps.

## 2. Overview of the Published Articles

This Special Issue includes seven research articles authored by international experts based on Poland (3 papers), Italy (1 paper), Spain (1 paper), UK & The Netherlands (1 paper), and China (1 paper).

The papers in this collection are organized around three key pillars of modern Heritage Science: Mechanical Degradation and Structural Integrity; Micro-Analytical Archaeometry and Provenancing; Affordable non-contact monitoring and characterization on site.

The advances in innovative testing procedures specifically tailored for cultural heritage have been illustrated, by adapting them to the peculiar characteristics of the historical artefacts. The reported research is focused on the knowledge of properties and performance of the historical materials, with attention also given to investigate the mechanism of decay and its progression over time.

In this latter context, dynamometric tensile tests on contemporary canvases (made of linen or polycotton), with and without ground layer, were used to develop a new methodology for quantifying the mechanical degradation under accelerated aging conditions [Contribution 1]. Differences in mechanical properties and behavior of the samples were found due to materials’ composition/nature and to the effects of accelerated aging. These results have been judged helpful to facilitate early detection of deterioration, as well as recognition of areas requiring treatment, both for well-timed conservative actions. Nevertheless, limitations to the effective application of the proposed method stemmed from the fact that a single-technique methodology was adopted.

The mechanical characterization of ancient materials faces a severe sampling bottleneck. Standard engineering tensile or compressive tests require large specimen volumes, which is a structural impossibility when dealing with fragile canvases, as in the above mentioned study, or historical masonry joints.

A minimally destructive testing technique, based on the double punch test (DPT), was employed for estimating the compressive strength of historical mortars [Contribution 2]; the samples were collected from four heritage brick structures in Krakow. The results found a relationship between compressive strength and bulk density, suggesting the latter parameter as a potential indicator of mortar strength. In addition, the method made it possible to identify the mortars from original constructions (generally having lower strengths) rather than the ones used in later reconstruction phases. Thus, using a general testing procedure (according to DIN 18555-9 code), standardized for hardened mortars in the bed joint, a tentative refinement methodology was proposed to assess the mechanical properties of mortars from heritage structures, where the sampling of large specimens is restricted and traditional destructive tests methods are infeasible.

Complementary methods are often used together to gain a comprehensive understanding of ancient materials’ composition. When evaluating complex organic residues or historical polychromies, a single technique rarely yields a complete picture. Multi-analytical protocols are proven to maximize diagnostic accuracy and to be more effective, especially for detecting unstable substances [8].

For example, in analyzing residues in ancient ceramic vessels, lipid compounds, even if in traces, are easily identifiable as they are quite stable [9]. Conversely, the determination of alkaloids in archaeological artefacts is difficult because these molecules are highly prone to degradation. Various environmental factors, such as temperature, humidity, pH, and microbial activity may affect the stability of the organic residues; contamination during the handling or recovery of artefacts can further complicate the analysis, leading to the detection of degradation products being considered as an alternative.

A dual-method analytical protocol [Contribution 3], based on chromatographic and spectroscopic methods (i.e., gas chromatography-tandem mass spectrometry (GC-MS/MS) and liquid chromatography-tandem mass spectrometry (LC-MS/MS)), allowed the verification of the presence of psychoactive opium and tropane alkaloids in pottery vessels and sherds associated with ritual contexts. Thus, analytical procedures that combine complementary techniques can increase detection reliability of organic residues in porous media. This can pave the way to wider applications in archaeometry, as well as in many other fields of materials science, such as pharmaceutical, food testing, environmental, medical, and forensic studies.

A multi-analytical approach has also been applied to study three polychrome wooden sculptures from Apulia, in Southern Italy, dated back to the Baroque period (17th–18th century) [Contribution 4]. In selecting the analytical methodologies, the preference was given to minimally invasive or non-destructive techniques which make it possible rapid analysis with limited sampling. Polarized light microscopy (PLM), in ultraviolet (UV) and visible (VIS) light, scanning electron microscopy equipped with EDS (SEM-EDS), Fourier-Transform Infrared (ATR-FTIR) spectroscopy and pyrolysis–gas chromatography/high-resolution accurate mass spectrometry (Py-GC/HRMS) were used. The comprehensive analytical protocol made it possible to identify the painting techniques used for the pictorial film and the ground preparation layers, thereby enabling differentiation between the artist’s original polychromies and repainting due to subsequent maintenance or restoration. The results of this study confirmed that integration of analytical data with art-historical and documentary studies can provide an added value to the knowledge of execution techniques, conservation, and restoration matters.

Data analysis is a fundamental pillar of data driven research and statistical methods in heritage science and can effectively step in to recognize patterns. The application of statistical methods to the analytical data helps to cluster the artefacts by provenance, typology, and date [10,11]. The latter details provide essential data for conservators, but they are also significant to enhancing the fruition of cultural heritage. In fact, the artefacts can be presented in exhibits and in museums, telling their story and their journey through time and countries.

A successful example of this approach to data analysis is the study of silver coins [Contribution 5] recovered from the shipwreck of the vessel “Rooswijk”, a ship used for many voyages, typically from Netherlands to Indonesia, that sank in 1740. The investigations, carried out using micro X-ray fluorescence (μXRF), and scanning electron microscopy combined with energy dispersive spectroscopy (SEM-EDS), provided a distinctive trace elemental fingerprint of the investigated items. The multi-analytical approach allowed researchers to recognize even the composition of coins poorly preserved because of corrosion. Statistical fingerprinting was achieved by applying Principal component analysis (PCA) to minor elements, such as Ni, Zn, Au and Bi, giving a reference tool for identifying the silver sources, mint location, and potential use of re-melted/recycled coins. Furthermore, the results of this study contributed to further knowledge about the global silver movement, coin circulation, and trade routes between the Americas, Europe, and Asia.

Affordability of the applied technologies is another current concern in the field of cultural heritage. As technology matures, there is an urgent push to develop affordable, highly reliable, and non-contact monitoring systems that can be easily deployed in situ. Moreover, diagnostic equipment is often expensive, difficult to access, and difficult to use, so research is focusing on developing highly mobile, user friendly, and cost-effective tools.

Low-cost LiDARs integrated into mobile devices have been proposed for 3D modelling, degradation assessing, as well as continuous monitoring of historical architectural elements [Contribution 6]. The use of handheld scanners and advanced apps has proved to be a practical and accessible solution. The effectiveness of such solutions depends on some aspects. In particular, size and shape of the object, scanning distance, used app, and post-processing were found key points affecting accuracy and helpfulness of the results. The proposed approach showed the advantages of mobility, affordability, and user-friendliness, but it seemed to be limited to investigating small sites; it has performed best on an area of about 1.5 m^2^, enabling the detection of cavities at least 2 cm wide and 1 cm deep. In handling large areas or complex structures, limitations due to significant errors were experienced; in these cases, traditional terrestrial laser scanning (TLS) still offers high precision and accuracy.

Non-destructive and non-contact procedures can take advantages from hyperspectral data acquisition systems which offer rapid, efficient, and precise methodologies for materials characterization [12]. Hyperspectral imaging captures optical information from continuous and narrow spectral bands reflected or emitted by a surface, thus allowing for accurate measurements over a broad range of wavelengths, from visible to infrared and ultraviolet light.

In pigment identification, hyperspectral imaging, which maps chemical composition across physical surfaces, represents the cutting edge and it has been used to investigate surface paintings in Buddhist caves of the Tang Dynasty in China [Contribution 7]. A comprehensive hyperspectral database has been developed, incorporating 24 common Chinese mineral pigments. The methodology applied in this study is in the early stages of development, and enhancements were achieved in terms of accuracy of pigment identification. In comparison to the commonly used databases, the precise identification of the pigments has included the spectral influence of binders, pigment particle sizes, and substrate.

## 3. Conclusions and Future Trends

The research compiled in this special issue spans several critical sub-fields of modern Heritage Science, including chemical fingerprinting, micro-mechanical testing, and digital spatial mapping. While each contribution resolves a distinct historical or material question, they show a collective trajectory toward non-destructive, multi-analytical, and cost-effective research framework, which define the trends reshaping 21st-century Heritage Science.

The articles collected demonstrate that Heritage Science has fully transitioned into an integrated, multi-scale discipline which is radically changing our ability to safeguard our shared past. By combining historical curiosity with state-of-the-art physics, chemistry, engineering, and digital spatial mapping, the scientific community can be better equipped than ever to study, restore, and monitor cultural artifacts; conservators can also be empowered to read the history of an object while designing data-backed preservation strategies.

The published studies address the pros and cons of contemporary investigation strategies, refine, or overcome existing technological limitations in the field; and the reported results point out that future research deserves to be directed toward:

- prioritizing minimally invasive techniques and diagnostic workflows;
- designing methods to be used for large-scale artworks;
- applying multi-modal technologies;
- optimizing experimental conditions to minimize data fluctuations due to environmental factors;
- developing standardized protocols;
- integrating multidisciplinary approaches.
